# Vitamin D Status and Atherogenic Lipid Profiles, Including Lipoprotein(a), in Elite Athletes

**DOI:** 10.3390/nu18122013

**Published:** 2026-06-21

**Authors:** Vincent Groesser, Astrid Most, Jamschid Sedighi, Priyanka Böttger, Samuel Sossalla, Pascal Bauer

**Affiliations:** 1 Department of Cardiology and Angiology, Justus-Liebig-University Giessen, 35390 Giessen, Germany; astrid.most@innere.med.uni-giessen.de (A.M.); jamschid.sedighi@gmail.com (J.S.); priyanka.boettger@uni-giessen.de (P.B.); samuel.sossalla@innere.med.uni-giessen.de (S.S.); pascal.bauer@innere.med.uni-giessen.de (P.B.); 2 Cardio-Pulmonary Institute (CPI), 35392 Giessen, Germany; 3 Department of Cardiology, Kerckhoff Clinic GmbH, 61231 Bad Nauheim, Germany

**Keywords:** vitamin D, 25-hydroxyvitamin D, LDL cholesterol, lipoprotein(a), elite athletes, lipid metabolism

## Abstract

**Background/Objectives:** Vitamin D has been implicated in lipid metabolism, but data regarding its association with atherogenic lipoproteins in elite athletes remain limited. Elite athletes represent a unique research model to investigate these associations with reduced confounding from obesity, chronic disease, smoking, and physical inactivity. **Methods:** This cross-sectional study included 773 male professional athletes from mixed sports disciplines (mean age 25.5 ± 5.0 years). Serum 25-hydroxyvitamin D [25(OH)D] concentrations and lipid parameters, including total cholesterol, low-density lipoprotein cholesterol (LDL-C), high-density lipoprotein cholesterol (HDL-C), triglycerides, and lipoprotein(a) [Lp(a)], were assessed. Associations were analyzed using correlation analyses, subgroup comparisons according to predefined 25(OH)D categories (<30, 30–50, and >50 ng/mL), and multivariable linear regression models adjusted for age, body mass index, season, and training-related variables. **Results**: Higher serum 25(OH)D concentrations were independently associated with lower LDL-C (*p* = 0.028), triglyceride (*p* = 0.002), and Lp(a) concentrations (*p* = 0.036), whereas no independent association was observed with HDL-C (*p* = 0.559). Athletes with 25(OH)D concentrations ≥30 ng/mL demonstrated lower LDL-C, triglyceride, and Lp(a) levels compared with athletes below this threshold (all *p* < 0.05). Higher vitamin D status was additionally associated with greater peak exercise performance (4.29 ± 1.15 vs. 3.36 ± 0.68 W/kg; *p* < 0.001). **Conclusions**: Higher 25(OH)D concentrations were independently associated with a more favorable lipid profile in elite athletes, including lower LDL-C, triglyceride, and Lp(a) concentrations. Prospective studies are warranted to further investigate the relationship between vitamin D status and lipid metabolism in athletic populations.

## 1. Introduction

Vitamin D has been implicated not only in calcium homeostasis and bone metabolism but also in several extra-skeletal processes, including immune regulation, inflammation, and metabolic health [1,2,3,4,5]. In this context, potential associations between circulating vitamin D levels—most commonly assessed as serum 25-hydroxyvitamin D [25(OH)D]—and lipid metabolism have attracted increasing interest [6,7,8].

In the general population, lower serum 25(OH)D concentrations have been reported to be associated with a less favorable lipid profile, including higher total cholesterol and low-density lipoprotein cholesterol (LDL-C) levels, as well as lower high-density lipoprotein cholesterol (HDL-C) concentrations [9]. In addition to traditional lipid fractions, lipoprotein(a) [Lp(a)] has emerged as an important genetically determined cardiovascular risk factor associated with lifelong atherosclerotic risk [10,11]. However, data regarding potential associations between vitamin D status and Lp(a), particularly in athletic populations, remain limited.

Proposed mechanisms linking vitamin D and lipid metabolism include vitamin D receptor-mediated regulation of gene expression, anti-inflammatory effects, and modulation of cholesterol metabolism [12]. However, findings across observational and interventional studies remain inconsistent, and the extent to which vitamin D status is independently associated with lipid parameters is still not fully understood. Meta-analyses of randomized controlled trials have reported divergent effects of vitamin D supplementation on lipid profiles, with some showing modest improvements in certain lipid fractions but others finding little or no impact on LDL-C and total cholesterol, highlighting the need for further research to clarify these relationships [13,14,15].

One major challenge in interpreting existing evidence lies in the presence of numerous confounding factors. Age, body composition, physical activity level, dietary habits, sun exposure, comorbidities, and medication use are all closely linked to both vitamin D status and lipid metabolism. In heterogeneous study populations, these factors may substantially influence observed associations and limit the ability to draw robust conclusions [16]. Consequently, it remains difficult to distinguish whether vitamin D itself is linked to lipid metabolism or primarily reflects broader lifestyle- and health-related factors.

Against this background, highly trained athletic populations may provide a unique physiological model for investigating early cardiometabolic phenotypes in otherwise healthy young individuals with low baseline cardiovascular risk. Due to their structured training routines, comparable lifestyle characteristics, and low prevalence of chronic disease, elite athletes may allow a more focused assessment of metabolic associations with reduced influence of major lifestyle-related confounders.

While vitamin D status has been increasingly studied in athletes, primarily in relation to musculoskeletal health and performance, data regarding its association with atherogenic lipid profiles in elite athletes remain limited [5,17]. Consequently, it remains difficult to distinguish whether vitamin D itself is linked to lipid metabolism or primarily reflects broader lifestyle- and health-related factors [18].

The present study therefore aimed to investigate the association between serum 25(OH)D concentrations and lipid parameters, including LDL-C, HDL-C, triglycerides, and Lp(a), in a well-defined cohort of male professional athletes from different team sports. By focusing on a young and physically active population with relatively homogeneous lifestyle characteristics, this study sought to examine early metabolic associations while minimizing the influence of major lifestyle-related confounders.

## 2. Methods

### 2.1. Study Design and Participants

This single-center cross-sectional registry study included professional athletes undergoing routine pre-season sports cardiology evaluation at the University Hospital of Giessen. Data were collected during standardized medical screening visits performed between 2014 and 2026. The cohort only included male professional athletes aged 18 to 39 years with available serum 25(OH)D measurements. Athletes without documented 25(OH)D levels were not included in the current analysis. Overall, 802 professional athletes underwent routine pre-season sports cardiology assessment during the study period. Twenty-nine athletes were excluded because of missing data on serum 25(OH)D concentrations and/or lipid parameters required for the present analyses. The final study population consisted of 773 male professional athletes (Supplementary Figure S1).

All tests were conducted at least 3 h post-prandially, and participants refrained from exercise for at least 36 h prior to testing.

### 2.2. Eligibility Criteria and Clinical Assessment

Only athletes without known cardiovascular, metabolic, or systemic disease and without regular medication use were eligible for inclusion. Information on medical history, medication use, training volume, and training history was obtained using standardized questionnaires.

### 2.3. Study Population Characteristics

The final study population consisted of 773 male professional athletes from various team sports disciplines (Handball, Basketball, Ice hockey, and Soccer) of the first and second divisions in Germany. All participants underwent standardized sports cardiology assessment including physical examination, blood pressure measurement, ECG, and echocardiography.

### 2.4. Laboratory Analyses

Venous blood samples were obtained from an antecubital vein with participants in a seated position and processed within 30 min in the certified central laboratory of the University Hospital of Giessen using standardized automated procedures. Serum 25(OH)D concentrations were measured as total 25-hydroxyvitamin D using a chemiluminescent immunoassay (LIAISON, DiaSorin, Saluggia, Italy). Separate measurements of 25(OH)D2 and 25(OH)D3 were not available. Parathyroid hormone was determined using an electrochemiluminescence immunoassay (Elecsys PTH [1–84], Roche Diagnostics, Mannheim, Germany). Serum lipid parameters, including total cholesterol, low-density lipoprotein cholesterol (LDL-C), high-density lipoprotein cholesterol (HDL-C), lipoprotein(a) [Lp(a)], and triglycerides, were measured using an automated chemistry analyzer (ADVIA, Siemens Healthineers, Erlangen, Germany) with Siemens reagents according to the manufacturer’s instructions and standard laboratory protocols. HbA1c, C-reactive protein, and serum calcium were measured using routine laboratory methods. Historical laboratory reports documented lipoprotein(a) concentrations below the assay detection threshold as “<10 mg/dL”. For statistical analyses, these values were assigned a value of 1 mg/dL to permit inclusion in continuous analyses and logarithmic transformation while avoiding zero values. A total of 164 participants (21.2%) had lipoprotein(a) concentrations reported as <10 mg/dL. In later assessments, exact values in the range of 0–10 mg/dL were available. Blood sampling was performed as part of routine preseason screening; fasting was not routinely required. We therefore treated lipid measurements as non-fasting. Given the postprandial variability of triglycerides, sensitivity analyses excluding triglycerides >4.5 mmol/L confirmed robustness of the findings.

Serum 25(OH)D concentrations were analyzed continuously and categorically using predefined thresholds (<30, 30–50, and >50 ng/mL) based on commonly used definitions of vitamin D sufficiency [19,20].

### 2.5. Training-Related Variables

Training history (years of professional training) and current training volume (hours per week) were recorded as part of the routine medical assessment. Maximal aerobic performance was assessed using standardized incremental cycle ergometry until volitional exhaustion. Peak performance was expressed as maximal achieved workload normalized to body weight (W/kg). These variables were included as training-related covariates.

### 2.6. Statistical Analysis

Continuous variables are presented as mean ± standard deviation (SD) or median (interquartile range [IQR]) as appropriate. Group comparisons were performed using independent-samples *t*-tests, one-way ANOVA, Mann–Whitney U tests, or Kruskal–Wallis tests where appropriate. Correlations between serum 25(OH)D concentrations and clinical parameters were assessed using Pearson or Spearman correlation analyses depending on data distribution.

Associations between serum 25(OH)D concentrations and lipid parameters were evaluated using multivariable linear regression analyses with separate models for each lipid outcome. All models were adjusted for age, body mass index (BMI), years of professional training, weekly training volume, peak performance (W/kg), sport discipline (handball, basketball, ice hockey, soccer), season of assessment (November–May vs. June–October), and assessment year. A predefined sensitivity analysis including only examinations performed between June and October was conducted to account for seasonal variation in vitamin D levels. Season was categorized as June–October and November–May to reflect expected differences in ultraviolet-B radiation exposure and cutaneous vitamin D synthesis in Central Europe. Peak exercise performance (W/kg) was included as a surrogate marker of overall physical fitness and training status.

Because of its right-skewed distribution, lipoprotein(a) was log-transformed prior to regression analyses. For log-transformed outcomes, back-transformed coefficients are presented as percent change per 1 ng/mL increase in serum 25(OH)D. Analyses were performed using complete-case data; therefore, sample sizes varied between variables.

Regression results are reported as unstandardized regression coefficients (β) with 95% confidence intervals (CIs). No adjustment for multiple comparisons was performed due to the exploratory nature of the analyses. A two-sided *p* value <0.05 was considered statistically significant. Statistical analyses were performed using JASP (version 0.9.1).

## 3. Results

### 3.1. Study Population and Vitamin D Status

A total of 773 male professional athletes were included, depending on data availability for individual variables. All athletes were actively competing at a professional level, had no chronic disease, and reported no regular medication use. The cohort included professional handball (n = 520), basketball (n = 57), ice hockey (n = 134), and soccer players (n = 62). Descriptive characteristics are summarized in Table 1.

Among athletes with available 25(OH)D measurements, examinations were conducted between July 2014 and January 2026. Most assessments occurred during June–October (n = 628; 81.2%), whereas 145 athletes (18.8%) were examined during November–May.

As expected, serum 25(OH)D concentrations were markedly higher during June–October compared with November–May (36.9 ± 13.2 vs. 22.9 ± 12.7 ng/mL; *p* < 0.0001). LDL-C, total cholesterol, and triglycerides did not differ significantly between seasons, whereas HDL-C was lower during November–May (47.4 ± 9.8 vs. 50.7 ± 10.8 mg/dL; *p* < 0.001). Lipoprotein(a) also differed modestly by season (*p* < 0.001). A sensitivity analysis restricted to summer assessments showed comparable associations between 25(OH)D and lipid parameters (Supplementary Table S1).

Serum 25(OH)D concentrations differed significantly across sports disciplines (Supplementary Table S2). Significant differences were also observed for total cholesterol, LDL-C, HDL-C, triglycerides, and lipoprotein(a) (all *p* < 0.05).

Based on the predefined cut-off of 30 ng/mL, athletes were categorized into vitamin D-insufficient (<30 ng/mL) and vitamin D-sufficient (≥30 ng/mL) groups.

### 3.2. Group Differences According to Vitamin D Status

Athletes with sufficient vitamin D status (≥30 ng/mL; n = 477) demonstrated a more favorable lipid profile than athletes with insufficient levels (<30 ng/mL; n = 296). Mean serum 25(OH)D concentrations were 42.25 ± 11.51 ng/mL and 21.36 ± 6.54 ng/mL, respectively (*p* < 0.001).

LDL-C concentrations were lower in athletes with sufficient vitamin D status (95.08 ± 27.77 vs. 101.06 ± 31.22 mg/dL; *p* = 0.006). Triglycerides were likewise lower (96.11 ± 47.69 vs. 105.96 ± 64.52 mg/dL; *p* = 0.020), and lipoprotein(a) concentrations differed significantly between groups (*p* = 0.048). In contrast, HDL-C did not differ significantly (*p* = 0.953), while total cholesterol showed only a trend toward lower values in the sufficient group (*p* = 0.052).

Age, BMI, training history, and weekly training volume were comparable between groups (all *p* > 0.05). Athletes with sufficient vitamin D status demonstrated higher peak exercise performance (4.29 ± 1.15 vs. 3.36 ± 0.68 W/kg; *p* < 0.001), lower parathyroid hormone concentrations (29.79 ± 15.70 vs. 40.14 ± 51.23 pg/mL; *p* < 0.001), and slightly higher serum calcium levels (2.36 ± 0.10 vs. 2.34 ± 0.12 mmol/L; *p* = 0.010). Detailed group characteristics are summarized in Table 2.

In an additional subgroup analysis, athletes were stratified into three categories according to serum 25(OH)D concentrations: <30 ng/mL (n = 296), 30–50 ng/mL (n = 389), and >50 ng/mL (n = 87). Age, BMI, training volume, and training history did not differ significantly across groups.

Increasing 25(OH)D concentrations were associated with progressively lower total cholesterol (*p* = 0.037), LDL-C (*p* = 0.004), and triglyceride levels (*p* = 0.022). The lowest LDL-C concentrations were observed in athletes with serum 25(OH)D >50 ng/mL (91.4 ± 25.96 mg/dL). Lipoprotein(a) levels also differed significantly across vitamin D strata (*p* = 0.047), although the distribution was non-linear. HDL-C levels did not differ significantly (*p* = 0.542).

Parathyroid hormone concentrations showed a marked inverse association with vitamin D status (*p* < 0.001). Peak aerobic performance increased across vitamin D categories, ranging from 3.36 ± 0.68 W/kg in athletes with <30 ng/mL to 4.73 ± 1.32 W/kg in those with >50 ng/mL (*p* < 0.001). Detailed subgroup results are presented in Table 3.

**Figure 1:**
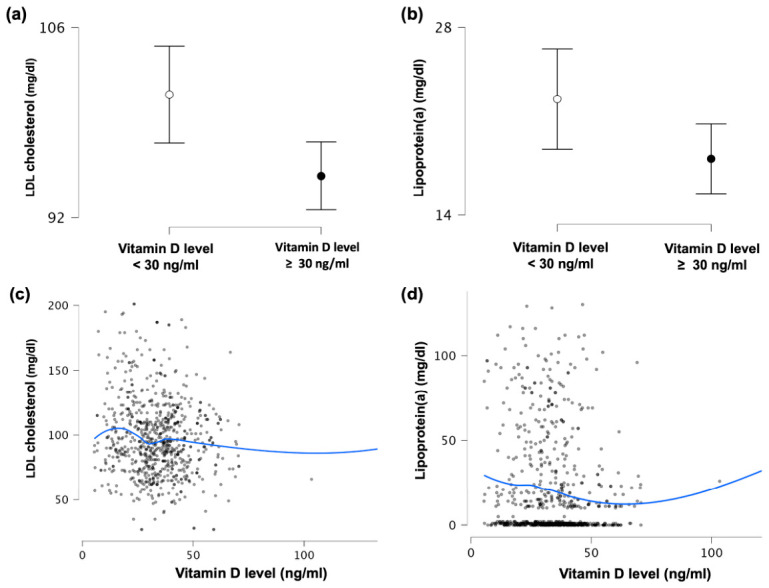
LDL-C and lipoprotein(a) concentrations according to categorical and continuous serum 25(OH)D levels. Mean ± SD/Median (IQR); LDL-C (**a**,**c**) and lipoprotein(a) (**b**,**d**) concentrations according to serum 25(OH)D status. (**a**,**b**) show concentrations stratified by serum 25(OH)D levels (<30 ng/mL and ≥30 ng/mL). (**c**,**d**) display the corresponding relationships across the continuous range of serum 25(OH)D concentrations using flexplots based on the raw data.

Figure 1 illustrates the relationships between serum 25(OH)D concentrations and both LDL-C and lipoprotein(a), using categorical and continuous representations of vitamin D status.

### 3.3. Univariate Correlations Between Serum 25(OH)D and Clinical Parameters

Serum 25(OH)D concentrations were inversely correlated with total cholesterol (r = −0.080; *p* = 0.034), LDL-C (r = −0.122; *p* < 0.001), triglycerides (r = −0.104; *p* = 0.006), and lipoprotein(a) (r = −0.120; *p* < 0.001). No significant association was observed with HDL-C (r = 0.061; *p* = 0.089).

In addition, serum 25(OH)D concentrations were inversely associated with HbA1c (r = −0.085; *p* = 0.019) and parathyroid hormone concentrations (r = −0.188; *p* < 0.001), while positively correlating with serum calcium levels (r = 0.108; *p* = 0.003).

No significant correlations were found between serum 25(OH)D concentrations and age, BMI, CRP, and weekly training volume. A weak but statistically significant correlation was observed with years of professional training. A strong positive correlation was observed between serum 25(OH)D concentrations and peak exercise performance (r = 0.440; *p* < 0.001). Correlation analyses are summarized in Table 4.

### 3.4. Multivariable Regression Analyses

Multivariable linear regression analyses adjusted for age, BMI, years of professional training, weekly training volume, peak performance, sport discipline, and season of assessment demonstrated independent inverse associations between serum 25(OH)D concentrations and several lipid parameters (Table 5).

Each 1 ng/mL increase in serum 25(OH)D was associated with a decrease in LDL-C of −0.19 mg/dL (95% CI −0.37 to −0.02; *p* = 0.028). Higher serum 25(OH)D concentrations were also independently associated with lower triglyceride levels (β = −0.57; 95% CI −0.92 to −0.22; *p* = 0.002).

For log-transformed lipoprotein(a), each 1 ng/mL increase in serum 25(OH)D was associated with a 1.1% decrease in Lp(a) concentrations (95% CI −2.1% to −0.1%; *p* = 0.036).

No independent association was observed between serum 25(OH)D concentrations and HDL-C in adjusted analyses.

**Figure 2:**
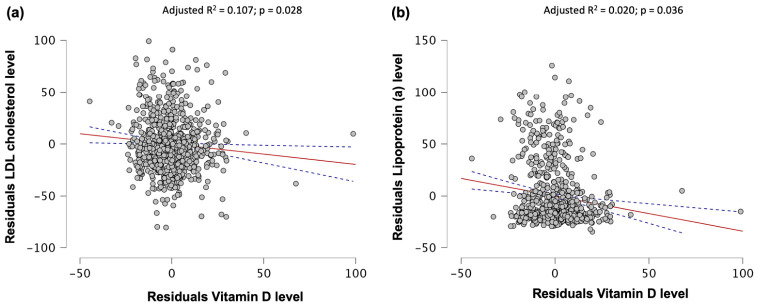
Adjusted association between serum 25(OH)D levels and LDL-C levels (**a**) as well as lipoprotein(a) (**b**). Scatter plots illustrating the associations between serum 25(OH)D concentrations and LDL-C (**a**) and lipoprotein(a) (**b**) after adjustment for age, BMI, training years, weekly training volume, peak performance (W/kg), sport discipline, and season of assessment. Solid red lines represent fitted regression lines and dashed blue lines indicate the corresponding 95% confidence intervals. Adjusted R^2^ values and *p*-values are displayed within each panel.

Associations between serum 25(OH)D concentrations and LDL-C as well as lipoprotein(a) are illustrated in Figure 2.

## 4. Discussion

In this large cohort of male professional athletes, higher serum 25(OH)D concentrations were associated with a more favorable lipid profile, including lower LDL-C, triglyceride, and lipoprotein(a) concentrations. These associations remained consistent after adjustment for anthropometric, training-related, and seasonal factors.

Serum 25(OH)D concentrations were categorized using the commonly applied threshold of 30 ng/mL for vitamin D sufficiency, consistent with previous studies in athletic populations and prior investigations from our group [21,22,23,24].

Extending this dichotomous classification, a three-level subgroup analysis revealed a graded association between serum 25(OH)D concentrations and lipid parameters. Athletes with serum 25(OH)D levels above 50 ng/mL exhibited the lowest concentrations of LDL-C and triglycerides, whereas lipoprotein(a) demonstrated a non-linear distribution across vitamin D strata. These findings may suggest that serum 25(OH)D concentrations above conventional sufficiency thresholds are associated with incrementally more favorable lipid profiles. This interpretation is consistent with previous expert statements and observational data suggesting potential extra-skeletal benefits of higher vitamin D concentrations, while acknowledging that optimal target levels remain under debate [19,25,26]. Importantly, these differences were observed in the absence of relevant differences in age, body composition, or training-related variables across vitamin D strata. As this analysis was exploratory, the results should be interpreted as hypothesis-generating.

Previous observational studies in the general population have linked low vitamin D status to adverse lipid profiles [8,27]. However, interpretation has often been limited by confounding from adiposity, physical inactivity, chronic disease, and medication use. In contrast, the present study was conducted in a highly selected cohort of elite athletes characterized by structured training routines, absence of major cardiometabolic disease, and limited variability in lifestyle-related factors. In addition, athletes from the same teams were generally assessed at the same preseason time points, further reducing environmental variability.

The additional sport-specific analyses should be interpreted in this context. Although statistically significant differences in serum 25(OH)D concentrations and lipid parameters were observed between sports disciplines, the corresponding effect sizes were generally modest, and all included disciplines were professional intermittent team sports with high cardiovascular and metabolic demands. Consistent with current European Society of Cardiology classifications, handball, basketball, ice hockey, and soccer are all considered mixed-type sports rather than fundamentally distinct athletic phenotypes [28]. Accordingly, sport discipline may capture a combination of calendar effects, indoor versus outdoor exposure, team-specific training and travel schedules, and behavioral factors related to sun exposure and supplementation rather than representing a primary determinant of the observed associations.

Nevertheless, residual confounding related to dietary patterns, supplement use, sun exposure, or socioeconomic factors cannot be totally excluded. In particular, information regarding vitamin D supplementation was not available in the present cohort. As vitamin D supplementation is common among elite athletes and may be associated with other health-promoting behaviors, part of the observed associations may reflect residual confounding rather than a direct biological effect of vitamin D itself. Therefore, the present findings should be interpreted as evidence of an association rather than proof of causality.

The persistence of associations after multivariable adjustment supports the hypothesis that vitamin D status may be independently related to lipid metabolism rather than simply reflecting differences in fitness or training load. Additional analyses restricted to summer assessments yielded comparable findings. Peak exercise performance was included as a surrogate marker of overall training status and physical fitness because both factors may influence serum 25(OH)D concentrations and lipid metabolism. However, it is also conceivable that vitamin D status may affect exercise performance, thereby placing performance on the causal pathway between vitamin D status and lipid outcomes. Consequently, adjustment for peak exercise performance may have resulted in partial over-adjustment and attenuation of the observed associations.

Several biological mechanisms could potentially explain the observed associations between vitamin D status and lipid metabolism. Experimental evidence suggests that vitamin D receptor signaling influences hepatic cholesterol homeostasis, inflammatory pathways, endothelial function, and insulin sensitivity, all of which are linked to atherogenic lipid metabolism and cardiovascular risk [12,29,30].

In addition, the close physiological relationship between vitamin D and parathyroid hormone (PTH) may be relevant in the present context. Elevated PTH concentrations have been associated with adverse metabolic and vascular effects in previous studies [31,32]. Although speculative, suppression of PTH by adequate vitamin D status could therefore represent another pathway linking vitamin D status to lipid metabolism.

A long-standing debate revolves around whether low vitamin D levels merely reflect poorer health status or reduced fitness rather than exerting independent metabolic effects. In the present cohort, however, the association between serum 25(OH)D and LDL-C persisted after adjustment for fitness- and training-related variables, supporting the possibility of a more direct metabolic influence.

From a clinical perspective, even modest differences in atherogenic lipid fractions during young adulthood may be biologically relevant, as cumulative exposure to atherogenic lipoproteins across the lifespan contributes to later cardiovascular risk (“cholesterol years”). Although elite athletes are generally considered metabolically healthy, dyslipidemia has also been reported in athletic populations [33]. In this context, the present findings may support the relevance of vitamin D status as a potential correlate of early metabolic phenotypes. Although causality cannot be inferred from this cross-sectional analysis, the observed associations support the hypothesis that vitamin D status may be linked to atherogenic lipid metabolism even in highly trained individuals with otherwise favorable metabolic profiles.

Similarly, the independent association between serum 25(OH)D and triglyceride concentrations is consistent with previous evidence linking vitamin D signaling to insulin sensitivity and hepatic lipid metabolism [34,35,36]. The detection of this association in professional athletes, who typically exhibit high insulin sensitivity and favorable metabolic profiles, underscores the robustness of the observed relationship.

The inverse association between serum 25(OH)D and lipoprotein(a) was particularly noteworthy. Lipoprotein(a) is largely genetically determined and only minimally modified by conventional lifestyle factors such as diet or physical activity. Evidence linking vitamin D status to lipoprotein(a) concentrations remains limited, and this relationship has not been systematically explored in healthy or athletic populations. Previous observational data in patients undergoing coronary angiography suggested that low vitamin D status and elevated lipoprotein(a) may cluster and jointly relate to coronary risk, [33], making the present findings in a young, physically active cohort of particular interest. However, the observed association should be interpreted cautiously. Given the strong genetic determination of lipoprotein(a) concentrations and their limited responsiveness to environmental or lifestyle-related influences, alternative explanations including residual confounding, ancestry-related differences, population stratification, and methodological aspects related to the handling of low lipoprotein(a) values cannot be excluded. Furthermore, the cross-sectional design precludes conclusions regarding causality or potential biological effects of vitamin D on lipoprotein(a) metabolism. Although mechanistic explanations remain speculative, potential effects on hepatic apolipoprotein(a) synthesis and inflammatory signaling pathways warrant further investigation.

Notably, no independent association was observed between serum 25(OH)D and HDL-C in adjusted analyses, consistent with previous studies, indicating that vitamin D status may be more closely related to atherogenic lipid fractions than to HDL-C [37].

Beyond lipid-related findings, higher serum 25(OH)D concentrations were associated with greater peak aerobic performance, consistent with previous investigations from our group in comparable athletic cohorts and previous literature describing associations between vitamin D status, muscle function, and athletic performance in elite athletes [38,39]. Although performance outcomes were not the primary focus of the present study, this observation further supports the broader physiological relevance of vitamin D status in elite athletes.

The present study extends our previous work, which primarily focused on the prevalence of vitamin D insufficiency and its associations with parathyroid hormone levels, vascular function, and performance-related parameters in professional athletes [21,22,23]. Using a substantially larger dataset and multivariable regression analyses, the current investigation extends these observations to lipid metabolism and provides new insights into potential cardiometabolic correlates of vitamin D status in elite athletes.

## 5. Limitations

Several limitations should be acknowledged when interpreting the present findings. First, the cross-sectional design precludes causal inference, and reverse causality cannot be excluded. In addition, selection bias related to the availability of serum 25(OH)D measurements cannot be ruled out. Vitamin D supplementation, dietary intake, body composition, and other lifestyle-related factors were not systematically assessed. Athletes with greater health awareness may therefore have been more likely to use supplements or follow dietary patterns associated with both higher vitamin D concentrations and more favorable lipid profiles, contributing to residual confounding.

Although all multivariable analyses were adjusted for season of assessment and assessment year, and additional sensitivity analyses restricted to summer assessments yielded comparable results, the distribution of assessments was not fully balanced across seasons. Furthermore, detailed quantification of ultraviolet exposure and outdoor training time was not available. However, the cohort consisted predominantly of indoor athletes, likely reducing variability related to sport-specific sunlight exposure. Although sports discipline was available and included in additional sensitivity analyses, residual confounding related to discipline-specific training characteristics, nutrition, or recovery practices cannot be completely excluded.

Blood sampling was performed under routine sports medical screening conditions, and fasting was not routinely required. This may particularly have influenced triglyceride measurements and contributed to additional variability in lipid parameters. Moreover, lipoprotein(a) concentrations are strongly influenced by genetic background; therefore, residual confounding related to ancestry cannot be fully excluded in the absence of detailed genetic or ethnic information. In addition, 164 participants (21.2%) had lipoprotein(a) concentrations reported below the assay detection threshold (<10 mg/dL). For statistical analyses, these values were assigned a value of 1 mg/dL. Although this approach enabled inclusion of all participants in continuous analyses, the handling of censored values may have influenced the observed associations involving lipoprotein(a).

Finally, the exclusive inclusion of male professional athletes limits the generalizability of the present findings to female athletes, recreational athletes, and non-athletic populations. Nevertheless, the standardized assessment procedures and relatively homogeneous lifestyle characteristics of this elite athletic cohort may have reduced variability related to major lifestyle-associated confounders. Furthermore, multiple statistical comparisons were performed without formal adjustment for multiplicity because the analyses were considered exploratory. Therefore, an increased risk of type I error cannot be excluded.

## 6. Conclusions

In this large and well-characterized cohort of male professional athletes, higher serum 25(OH)D concentrations were independently associated with a more favorable atherogenic lipid profile, including lower LDL-C, triglyceride, and lipoprotein(a) concentrations. These associations remained consistent after adjustment for anthropometric, training-related, and seasonal factors, suggesting that vitamin D status may represent a relevant correlate of lipid metabolism in this athletic population with relatively homogeneous lifestyle characteristics and low burden of cardiometabolic confounding factors.

Exploratory subgroup analyses further suggested graded associations between serum 25(OH)D concentrations and atherogenic lipid parameters, with more favorable lipid profiles observed at higher vitamin D levels. Although these findings do not support specific treatment targets, they raise the hypothesis that conventional vitamin D sufficiency thresholds may not necessarily reflect optimal concentrations for cardiometabolic health in elite athletes.

Given the recognized importance of cumulative exposure to atherogenic lipoproteins across the lifespan, even modest differences in lipid profiles during young adulthood may be biologically relevant. While the cross-sectional design precludes causal inference, the consistency of associations across different lipid fractions and analytical approaches supports further investigation into vitamin D status as a potential metabolic correlate in athletes. Prospective and interventional studies are warranted to clarify the directionality and potential physiological relevance of these associations.

## Figures and Tables

**Table 1:** Descriptive characteristics of the study population (descriptives as mean ± SD/median (IQR).

	Mean ± SD/Median (IQR)	n
Age (years)	25.54 ± 4.97	773
BMI (kg/m^2^)	25.50 ± 2.08	773
25(OH)D (ng/mL)	34.25 ± 14.19	773
Total cholesterol (mg/dL)	162.61 ± 30.48	713
LDL cholesterol (mg/dL)	97.65 ± 29.27	773
HDL cholesterol (mg/dL)	50.19 ± 10.68	773
Triglycerides (mg/dL)	99.73 ± 54.86	712
Lipoprotein(a) (mg/dL)	1.00 (0.00–24.00)	773
HbA1c (%)	5.24 ± 0.26	773
CRP (mg/L)	0.67 ± 2.07	773
Training volume (min/week)	1150.96 ± 235.77	764
Training history (years)	8.62 ± 5.05	764
Peak performance (watts/kg)	3.93 ± 1.08	773

**Table 2:** Group differences according to sufficient or insufficient Vitamin D status (descriptives as mean ± SD/median (IQR).

	25(OH)D < 30 ng/mL (n = 296)	25(OH)D ≥ 30 ng/mL (n = 477)	*p*
Age (years)	25.35 ± 5.03	25.62 ± 4.97	0.473
BMI (kg/m^2^)	25.54 ± 2.20	25.47 ± 2.00	0.649
25(OH)D (ng/mL)	21.36 ± 6.54	42.25 ± 11.51	<0.001 *
Total cholesterol (mg/dL)	165.33 ± 32.00	160.76 ± 29.48	0.052
LDL cholesterol (mg/dL)	101.06 ± 31.22	95.08 ± 27.77	0.006 *
HDL cholesterol (mg/dL)	50.15 ± 11.94	50.10 ± 9.83	0.953
Triglycerides (mg/dL)	105.96 ± 64.52	96.11 ± 47.69	0.020 *
Lipoprotein(a) (mg/dL)	2.00 (1.00–39.00)	1.00 (0.00–23.00)	0.003
HbA1c (%)	5.26 ± 0.25	5.22 ± 0.26	0.057
CRP (mg/L)	0.622 ± 2.00	0.70 ± 2.13	0.610
Calcium (mmol/L)	2.34 ± 0.12	2.36 ± 0.10	0.010 *
Parathyroid hormone (pg/mL)	40.14 ± 51.23	29.79 ± 15.70	<0.001 *
Training volume (h/week)	19.36 ± 4.01	19.16 ± 3.83	0.500
Training history (years)	8.25 ± 5.07	8.88 ± 5.04	0.099
Peak performance (watts/kg)	3.36 ± 0.68	4.29 ± 1.15	<0.001 *

* Statistically significant (*p* < 0.05).

**Table 3:** Group differences according to insufficient (<30 ng/mL), sufficient (30–50 ng/mL) or optimal (>50 ng/mL) Vitamin D status (descriptives as mean ± SD/median (IQR)).

	25(OH)D < 30 ng/mL (n = 296)	25(OH)D 30–50 ng/mL (n = 389)	25(OH)D > 50 ng/mL (n = 87)	*p*
Age (years)	25.35 ± 5.03	25.59 ± 5.01	25.44 ± 4.50	0.918
BMI (kg/m^2^)	25.54 ± 2.20	25.41 ± 1.95	25.77 ± 2.20	0.296
25(OH)D (ng/mL)	21.36 ± 6.54	38.33 ± 5.13	60.23 ± 14.48	<0.001 * (a, b, c)
Total cholesterol (mg/dL)	165.33 ± 32.00	160.85 ± 29.27	157.95 ± 26.79	0.037*
LDL cholesterol (mg/dL)	101.06 ± 31.22	95.53 ± 27.69	91.40 ± 25.96	0.004 * (a, b)
HDL cholesterol (mg/dL)	50.15 ± 11.94	49.77 ± 9.63	51.10 ± 10.62	0.542
Triglycerides (mg/dL)	105.96 ± 64.52	97.44 ± 47.90	89.15 ± 45.42	0.022 * (b)
Lipoprotein(a) (mg/dL)	2.00 (1.00–39.00)	1.00 (0.00–24.00)	11.00 (0.00–22.00)	0.047 *
HbA1c (%)	5.26 ± 0.25	5.23 ± 0.26	5.18 ± 0.30	0.051
CRP (mg/L)	0.62 ± 2.00	0.64 ± 1.83	0.97 ± 3.16	0.368
Calcium (mmol/L)	2.34 ± 0.12	2.35 ± 0.10	2.39 ± 0.11	0.002 * (b, c)
Parathyroid hormone (pg/mL)	40.14 ± 51.23	30.84 ± 15.77	25.01 ± 14.50	<0.001 * (a, b)
Training volume (h/week)	19.36 ± 4.01	19.03 ± 3.90	19.85 ± 3.39	0.193
Training history (years)	8.25 ± 5.07	8.77 ± 5.11	9.06 ± 4.26	0.407
Peak performance (watts/kg)	3.36 ± 0.68	4.19 ± 1.01	4.73 ± 1.32	<0.001 * (a, b, c)

a = significant difference between <30 ng/mL and 30–50 ng/mL. b = significant difference between <30 ng/mL and >50 ng/mL. c = significant difference between 30–50 ng/mL and >50 ng/mL. * Statistically significant (*p* < 0.05).

**Table 4:** Correlations between serum 25(OH)D and clinical parameters.

	r (95–CI)	*p*
Age (years)	0.042 (−0.029–0.112)	0.243
BMI (kg/m^2^)	0.012 (−0.058–0.083)	0.735
Total cholesterol (mg/dL)	−0.080 (−0.152–−0.006)	0.034 *
LDL cholesterol (mg/dL)	−0.122 (−0.191–−0.052)	<0.001 *
HDL cholesterol (mg/dL)	0.061 (−0.009–0.131)	0.089
Triglycerides (mg/dL)	−0.104 (−0.176–−0.030)	0.006 *
Lipoprotein(a) (mg/dL)	−0.120 (−0.190–−0.050)	<0.001 *
HbA1c (%)	−0.085 (−0.155–−0.014)	0.019 *
CRP (mg/L)	0.037 (−0.033–0.108)	0.301
Calcium (mmol/L)	0.108 (0.037–0.177)	0.003 *
Parathyroid hormone (pg/mL)	−0.188 (−0.255–−0.118)	<0.001 *
Training volume (h/week)	0.012 (−0.060–0.084)	0.738
Training history (years)	0.089 (0.017–0.160)	0.015 *
Peak performance (watts/kg)	0.440 (0.381–0.495)	<0.001 *

* Statistically significant (*p* < 0.05).

**Table 5:** Associations between serum 25(OH)D concentrations and lipid parameters derived from multivariable linear regression analyses.

Lipid Parameter	β (95–CI) per 1 ng/mL Increase in 25(OH)D	*p*
Cholesterol	−0.231 (−0.419–−0.043)	0.016 *
LDL cholesterol	−0.194 (−0.368–−0.021)	0.028 *
HDL cholesterol	−0.020 (−0.085–0.046)	0.559
Triglycerides	−0.568 (−0.919–−0.216)	0.002 *
Lipoprotein(a)	−0.011 (−0.021–−0.001)	0.036 *

* Statistically significant (*p* < 0.05).

## Data Availability

The data presented in this study are not publicly available due to data protection regulations and ethical restrictions related to the use of pseudonymized clinical data from professional athletes. Data may be made available by the corresponding author upon reasonable request and subject to approval by the responsible ethics committee and applicable data protection requirements.

## Supplementary Materials

The following supporting information can be downloaded at https://www.mdpi.com/article/10.3390/nu18122013/s1, Table S1: Group differences according to insufficient (<30 ng/mL), sufficient (30–50 ng/mL) or optimal (>50 ng/mL) Vitamin D status–summer examinations (June–October) only (descriptives as mean ± SD/median (IQR)); Figure S1: Flow chart of participant selection; Table S2: Vitamin D status and lipid profile according to sports discipline (descriptives as mean ± SD/median (IQR).
