# Supplementary material for: Vitamin D Status and Atherogenic Lipid Profiles, Including Lipoprotein(a), in Elite Athletes
Source: Nutrients. 2026 Jun 21;18(12):2013. doi: 10.3390/nu18122013 (PMC13305039; doi:10.3390/nu18122013)
Supplement: Supplementary file 1 [file nutrients-18-02013-s001.zip › nutrients-4374482-supplementary.pdf]

Supplementary material

Supplementary Table S1: Group differences according to insufficient (<30 ng/ml), sufficient (30-50 ng/ml) or optimal (>50 ng/ml) Vitamin D status – summer examinations (June – October) only (*descriptives as mean ± SD / median (IQR))*

	25-OH-Vitamin D <30ng/ml (n=296)	25-OH-Vitamin D 30-50ng/ml (n=389)	25-OH-Vitamin D >50ng/ml (n=87)	p
Age (years)	25.30 ± 5.10	25.46 ± 4.85	25.37 ± 4.44	0.935
BMI (kg/m^2)	25.62 ± 2.25	25.48 ± 1.90	25.68 ± 2.01	0.598
25-OH-Vitamin D (ng/ml)	23.89 ± 5.14	38.28 ± 5.07	60.52 ± 14.91	<0.001* (a, b, c)
Total cholesterol (mg/dl)	169.90 ± 33.68	160.98 ± 29.78	157.53 ± 27.61	0.002*
LDL cholesterol (mg/dl)	101.17 ± 32.47	95.30 ± 27.92	91.41 ± 26.54	0.020* (a, b)
HDL cholesterol (mg/dl)	52.24 ± 12.91	49.98 ± 9.58	50.67 ± 10.23	0.066
Triglycerides (mg/dl)	111.73 ± 71.99	98.66 ± 49.18	89.38 ± 46.06	0.008* (a, b)
Lipoprotein(a) (mg/dl)	1.00 (0.00 – 43.00)	1.00 (0.00 – 24.50)	10.00 (0.00 – 18.00)	0.013* (a, b)
HbA1c (%)	5.24 ± 0.26	5.23 ± 0.25	5.18 ± 0.30	0.205
CRP (mg/l)	0.64 ± 2.10	0.59 ± 1.75	0.72 ± 2.33	0.858
Calcium (mmol/l)	2.34 ± 0.13	2.36 ± 0.10	2.39 ± 0.11	0.008* (b, c)
Parathyroid hormone (pg/ml)	41.79 ± 62.80	30.97 ± 16.03	25.21 ± 14.70	<0.001* (a, b)
Training volume (h/week)	18.70 ± 4.49	18.73 ± 3.36	19.60 ± 3.16	0.150
Training history (years)	8.38 ± 5.25	8.67 ± 5.03	9.01 ± 0.49	0.627
Peak performance (watts/kg)	3.49 ± 0.82	4.08 ± 1.19	4.66 ± 1.26	<0.001* (a, b, c)

a = significant difference between <30ng/dl and 30-50ng/dl

b = significant difference between <30ng/dl and >50ng/dl

c = significant difference between 30-50ng/dl and >50ng/dl

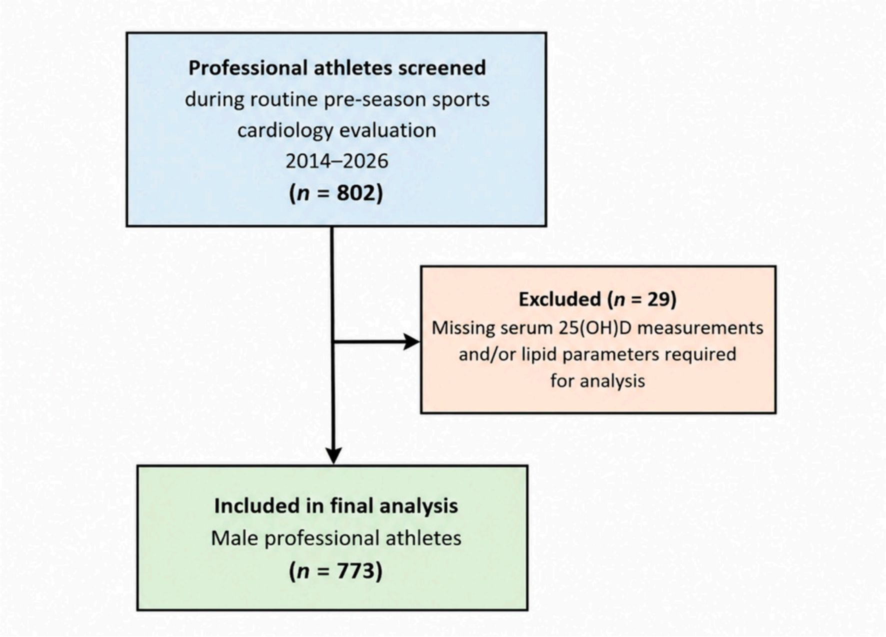

Supplementary Figure S1. Flow chart of participant selection

Supplementary Table S2. Vitamin D status and lipid profile according to sports discipline *(descriptives as mean ± SD / median (IQR)*

Variable	Handball (n = 520)	Basketball (n = 62)	Ice hockey (n = 134)	Soccer (n = 57)	p-value
25(OH)D (ng/mL)	34.47 ± 13.98	28.90 ± 11.56	37.72 ± 14.37	29.90 ± 15.55	<0.001
Total cholesterol (mg/dL)	165.17 ± 29.72	153.75 ± 29.57	162.98 ± 33.80	151.89 ± 25.93	0.002
LDL-C (mg/dL)	99.50 ± 29.33	88.42 ± 31.55	99.07 ± 28.94	88.48 ± 23.95	0.001
HDL-C (mg/dL)	49.77 ± 9.43	54.17 ± 11.62	49.75 ± 14.36	50.62 ± 9.88	0.021
Triglycerides (mg/dL)	107.05 ± 59.14	81.51 ± 42.31	94.96 ± 44.64	74.37 ± 38.40	<0.001
Lipoprotein(a) (mg/dL), median (IQR)	1.00 (0.00–24.00)	1.00 (0.25–36.25)	11.00 (1.00–29.00)	1.00 (1.00–17.75)	0.004
